# Large language models for prognostic analysis in mechanical fault diagnosis

**DOI:** 10.1371/journal.pone.0337203

**Published:** 2025-11-21

**Authors:** Hao Zhang, Wei Wang, Longfei Zhang, Siyu Shao, Qingli Wang, Jiandong Li, Jun Hu

**Affiliations:** 1 The graduate school of Air Force Engineering University, Xi’an, China; 2 Air and Missile Defense College of Air Force Engineering University, Xi’an, China; Xi'an Jiaotong University, CHINA

## Abstract

With the in-depth development of industrial intelligence, as the core basic component of high-end equipment, the fault diagnosis and health management of rotating machinery has become a key link to ensure the reliability of complex systems. Although the intelligent diagnosis technology based on mechanical vibration signals has made remarkable progress, in complex mechanical systems, it is difficult to comprehensively cover the fault feature space using vibration signal data only.This paper proposes an intelligent diagnosis framework based on a large language model. By empowering the large language model through multimodal data feature fusion and constructing a ternary data system of “raw vibration signals - time-frequency spectrum features - fault knowledge text”, the framework realizes cross-modal joint representation of mechanical fault features and breaks through the bottlenecks of traditional methods, such as insufficient feature extraction capability under complex working conditions and limited cross-scenario generalization. The framework innovatively integrates the deep semantic understanding ability of pre-trained large language models with mechanical fault mechanisms. Through the method of plugging in principle knowledge bases, the model can not only output fault location results but also simultaneously generate interpretable reports including fault cause analysis and maintenance strategy suggestions.The model proposed in this paper has been strictly tested on bearing datasets. Experimental results demonstrate that the model exhibits excellent performance and adaptability in different industrial scenarios.

## Introduction

With the development of Industry 4.0 and intelligent manufacturing, rotating machinery act as the core component of key fields such as energy and power, aerospace, and high-end manufacturing. Its reliability is directly related to the operational efficiency and the safety of industrial systems [1,2]. According to statistics from the Global Equipment Maintenance Association, unplanned shutdowns caused by rotating machinery failures result in annual losses of over $500 billion for global industrial enterprises, with 40% of these losses stemming from the ineffective identification of early-stage faults. Traditional monitoring and management models based on threshold alarms and periodic inspections have struggled to meet the urgent needs of modern industry for high reliability and low maintenance costs, due to the complex fault mechanisms and dynamic working conditions. Prognostic and Health Management (PHM) technology provides a theoretical framework for achieving predictive maintenance through real-time monitoring, condition assessment, and remaining useful life prediction [3,4].

Early fault detection methods mostly relied on manually accumulated experience for fault diagnosis. However, with the continuous development and improvement of sensor technology and fault diagnosis technology, relying solely on manual experience can no longer meet the needs of fault diagnosis. Vibration detection method extracts vibration signals through sensors, then extracts the time-frequency domain feature information contained in the vibration signals, and finally compares it with the frequency of fault features to further determine the specific location of the fault [5–7]. Due to its simple operation and convenient collection of vibration signals, it is more favored by researchers compared with other methods and has become the mainstream method in traditional fault diagnosis. Although researchers can diagnose various faults by extracting the feature information contained in the signals, this has not fundamentally solved the problem. Feature extraction still requires manual operation, and when the signal is mixed with noise, the credibility of the extracted features will be greatly reduced, leading to misjudgment in fault diagnosis.

Since AlexNet’s breakthrough in the ImageNet competition in 2012 [8], artificial intelligence technologies represented by deep neural networks have completely reshaped the research paradigm of rotating machinery fault diagnosis. Vibration signals, as the “gold standard” for condition monitoring of rotating machinery, have undergone a revolutionary leap in processing technology from shallow models to deep architectures [9]. A large number of deep learning models have been applied in the field of fault diagnosis, such as AutoEncoders (AE) [10], Deep Belief Networks (DBN) [11], Convolutional Neural Networks(CNN) [12], Recurrent Neural Networks (RNN) [13], and Generative Adversarial Networks(GAN). Fault diagnosis methods combined with deep neural networks can adaptively extract features from vibration signals through model training, replacing the steps of feature extraction, selection, and fault classification in traditional fault diagnosis methods. This saves a lot of human resources and achieves better fault diagnosis results than traditional methods.

However, since industrial equipment usually operates in a normal state, some fault conditions are difficult to collect, resulting in a serious shortage of fault data. In such cases, the diagnostic performance of fault diagnosis methods based on deep neural networks will be greatly reduced because there are not enough samples for model training. Therefore, in the field of fault diagnosis, research on transfer learning has also developed rapidly in recent years [14,15]. The essence of transfer learning is to use data from other working conditions similar to the current working condition as the data for model training, and transfer the knowledge identified and learned in one domain to another domain, thereby solving the problem of insufficient sample data in specific domains.

Both deep learning and transfer learning technologies face numerous challenges in the field of fault diagnosis: for example, information from a single sensor is difficult to comprehensively capture complex fault features, and the generalization ability of learning models is significantly restricted by changes in working conditions. In recent years, breakthroughs in pre-trained Large Language Model (LLM) in the fields of natural language and computer vision have demonstrated excellent transfer learning and contextual understanding capabilities, bringing revolutionary opportunities to the field of fault diagnosis. Large language models can help systems better analyze and utilize various types of data, achieve more natural human-computer interaction, and enhance the system’s knowledge ability as well as predictive and diagnostic capabilities.

It is crucial to clearly recognize that the development of large models in this domain remains in its early stages, with several technical challenges yet to be overcome. For instance, there exists an inherent discrepancy between time-series data like vibration signals and the textual semantic space where language models excel. Existing cross-modal alignment methods tend to lose critical physical features in the process. Moreover, single-modal vibration signal data cannot comprehensively characterize fault information, while significant variations in vibration signal characteristics under different loads and rotational speeds lead to insufficient generalization capability of large models for cross-operating-condition fault diagnosis.

Taking the above issues into consideration, this paper proposes an intelligent diagnostic framework based on multimodal large models. Different from existing fault diagnosis methods using large language models, we integrate three types of modal data: original vibration time-domain signals, time-frequency spectrograms, and fault knowledge texts. This enables the model to extract fault features more accurately and achieve more precise fault diagnosis. Experimental verification on a large number of public datasets has shown that our method is reasonable and effective. Overall, this paper makes the following contributions:

1. Through modal alignment, the digital features of vibration signals are aligned with the semantic space of text knowledge, enabling large language models to effectively understand non-text features and realize the joint representation of cross-modal data.
2. The complementarity of multimodal data is utilized to make up for the defects of a single sensor under complex working conditions, effectively improving the model’s ability in fault diagnosis under cross-operating conditions.
3. Through an innovative approach that incorporates an external principle knowledge base, the framework effectively combines the deep semantic understanding capabilities of pre-trained large language models with the failure mechanisms of rotating machinery. This enables the model not only to output fault localization results but also to simultaneously generate interpretable reports on root cause analysis and maintenance strategy recommendations.

The remaining part of this paper is organized as follows: The Related Work Section reviews existing research on fault diagnosis. The Methodology Section elaborates on the proposed method, and the Experiments Section discusses the experimental results and analysis, including ablation experiments, cross-operating-condition dataset validation and so on. Finally, the Conclusions Section summarizes the entire paper.

## Related work

### A. Fault diagnosis based on deep neural network

With the increasing complexity of rotating machinery systems and the rapid development of sensing technologies, a large amount of condition monitoring data has been collected, presenting big data characteristics such as diversity, nonlinearity, and variability [16], which has pushed the fault diagnosis technology of rotating machinery into the “big data” era [17]. The intelligent fault diagnosis method based on deep neural networks can automatically extract more discriminative fault features from data by constructing neural network models with deeper structures and stronger nonlinear fitting capabilities, and establish the mapping relationship between features and bearing health status, ultimately realizing an end-to-end fault diagnosis process. Compared with the diagnosis process based on shallow machine learning, it does not require manual participation in feature extraction and screening, which not only avoids the possible one-sidedness of manually extracted features but also significantly improves the efficiency and accuracy of model construction.

In recent years, deep learning-based fault diagnosis methods have become the mainstream research direction in intelligent fault diagnosis of rotating machinery, with a large number of research results emerging. Vo T et al. integrated one-dimensional convolutional neural networks (1D-CNN) and RNN into two independent processing streams. In this way, spatial features, temporal features, long-term features, and short-term features can be extracted from the original input signals. The multi-head mechanism is used to make the model focus more on relevant features and prevent overfitting, providing a practical and cost-effective solution for real-time fault detection in industrial applications [18]. Chen et al. developed a bearing fault diagnosis method based on Stacked Denoising AutoEncoders (SDAE). In terms of fault information acquisition, two vibration sensors are used to collect vibration data of bearings from two directions to enrich fault information. In terms of diagnostic network design, a new comprehensive loss function in the reverse fine-tuning process of SDAE is proposed, which makes network optimization more conducive to feature classification, providing a more reliable solution for bearing fault diagnosis in complex industrial scenarios [19]. Xu et al. fused CNN, GRU, and Attention Mechanism to construct various diagnostic models, and the proposed method significantly reduces the computation time and can effectively identify the changing trends of probability values in different iterations [20].

Although deep learning-based fault diagnosis methods have shown strong capabilities in condition monitoring and fault identification, limited by technical characteristics and application scenarios, there are still some significant problems to be solved. For example, the training of deep learning models requires a large amount of data with clear fault labels, but fault data is often scarce. If the distribution of training data is uneven, overfitting is very likely to occur.

#### B. Fault diagnosis based on LLM.

Large language models are renowned for their impressive reasoning capabilities in natural language processing tasks. Trained on massive amounts of text data, they can handle various natural language tasks such as text generation, text summarization, sentiment analysis, and question-answering systems. In recent years, large language models have developed rapidly, and there have been a small number of applications in the field of fault diagnosis. Zhang et al. [21] designed a prompt structure specifically for time-series data fault diagnosis, using large language models for fault detection, which has promoted process autonomy in the iron and steel metallurgy sector and enhanced human-machine collaboration. Tao et al. [22] proposed an LLM-based bearing fault diagnosis framework, which solves the problem of extracting semantic information from vibration data through joint extraction of time-frequency domain features based on a statistical analysis framework, filling the research gap in the application of LLMs in bearing fault diagnosis. Alsaif et al. [23] proposed a novel multimodal large language model fault detection and diagnosis framework, which uses large language models to synthesize datasets for knowledge base expansion and employs fine-tuning to improve the model scalability, generalization ability, and efficiency in handling complex systems and various complex fault scenarios.

Currently, the application of LLMs in the field of fault diagnosis still faces challenges. On one hand, industrial scenarios have high requirements for the “interpretability” of fault diagnosis results, yet the black-box nature of LLMs may make it difficult to trace diagnostic conclusions. On the other hand, the hallucination problem of large models can lead to the generation of false content, which poses significant potential risks in the field of fault diagnosis. Industrial fault diagnosis relies not only on time-series data such as vibration and temperature collected by sensors, but also on textual information including equipment manuals, maintenance records, expert experience documents, and fault case databases. Traditional deep learning models struggle to connect such “data” with “knowledge”, while large language models, by virtue of their ability to understand professional texts, hold great application prospects.

## Methodology

### A. Overall architecture

Consistent with common usage methods of LLM, we treat the discrimination of rotating machinery fault diagnosis as an LLM question-answering task. To better address issues such as the singularity of fault data features, poor model generalization ability, and hallucination, we adopt multiple key modules to cooperate with the powerful reasoning capability of LLM, thereby effectively achieving the accuracy of fault diagnosis.

**Fig 1:**
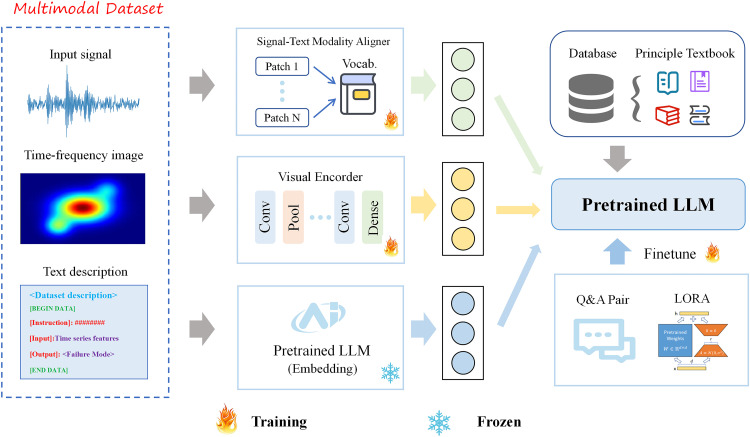
Overall perspective of fault diagnosis framework.

As shown in Fig 1, the proposed intelligent fault diagnosis framework utilizes three types of input data to identify the type of rotating machinery faults, assess fault severity, and provide reasonable maintenance recommendations, ultimately generating textual descriptions. The model architecture consists of five key components.

**Signal-Text Modality Aligner.** It is one of the core modules capable of realizing cross-modal information conversion. This component aligns words describing signal characteristics with time-series segments, thereby establishing semantic associations between them and providing underlying support for the conversion of signal features into interpretable text features. At the level of the core alignment mechanism, the module adopts a multi-head attention mechanism to linearly combine words. Different from the traditional single-head attention, this mechanism deploys multiple attention “heads” in parallel to perform multi-scale matching between the high-dimensional semantic vectors of dictionary words and the feature matrix of time series, mining the hidden relationships between cross-modalities, and establishing corresponding relationships for time-series segments, thus realizing the conversion of signal features into semantic text features. This module converts the abstract digital features in the signal into human-understandable natural language expressions, providing an intuitive semantic basis for subsequent applications such as signal analysis and fault diagnosis.

**Visual Encoder.** In mechanical fault diagnosis, time-frequency imaging technology is a commonly used technical method. In this paper, vibration signals are converted to the frequency domain through Continuous Wavelet Transform (CWT), thereby transforming sensor data into two-dimensional images and providing an understanding of the temporal dimension of the original data. As a core module connecting time-frequency images and text information, the Visual Encoder module adopts a deep learning architecture to encode the input two-dimensional time-frequency images. It extracts local features of the images through convolutional layers, suppresses noise interference using residual connections and normalization operations, and maps high-dimensional image feature vectors to the semantic space compatible with large language models via linear projection layers, ensuring effective matching between visual features and linguistic features.

**Embedding module.** The role of the Embedding module is to convert discrete raw fault text data into associated semantic vectors, such that the distance between vectors can reflect semantic relevance. For example, the vector distance between “bearing” and “gear” is closer than that between “bearing” and “voltage” because they have stronger semantic relevance in the field of mechanical fault diagnosis. The Embedding module adopts pre-trained models with relatively small parameter amounts, such as Bidirectional Encoder Representations from Transformers (BERT) and Contrastive Language-Image Pretraining (CLIP), to extract text and image features. Meanwhile, it converts texts such as professional textbooks and fault cases into vectors and stores them in the knowledge base, providing support for subsequent matching retrieval and content generation by LLM.

**Fine-Tuning Module.** The Fine-Tuning Module is the core module for converting general LLM into professional large model in the field of fault diagnosis. Through targeted training with data from the fault diagnosis domain, it enables the vertical application of the model. Due to the enormous number of parameters in LLM, we employ parameter-efficient fine-tuning techniques during the training process. By freezing most of the parameters of the pre-trained model and using the Low-Rank Adaptation (LoRA) method to update only a small number of parameters, we not only reduce training costs but also avoid catastrophic forgetting of the model caused by limited data volume.

**RAG-Enhanced Retrieval Module.** Retrieval Augmented Generation (RAG) technology is to supplement large language models with domain-specific knowledge. This preprocesses fault principle textbooks by segmenting them into semantically coherent text chunks—either by chapter/paragraph or sliding window—and converts them into vectors containing professional semantic information using a pre-trained model. This constructs a fault knowledge vector database that supports rapid semantic matching.

Through the coordinated interaction of these modules, the proposed model achieves seamless integration among fault vibration signals, images, and linguistic features, thereby enhancing its performance in generating relevant and precise textual outputs for fault diagnosis tasks.

### B. Components of the proposed framework

1. Signal-Text Modality Aligner

**Fig 2:**
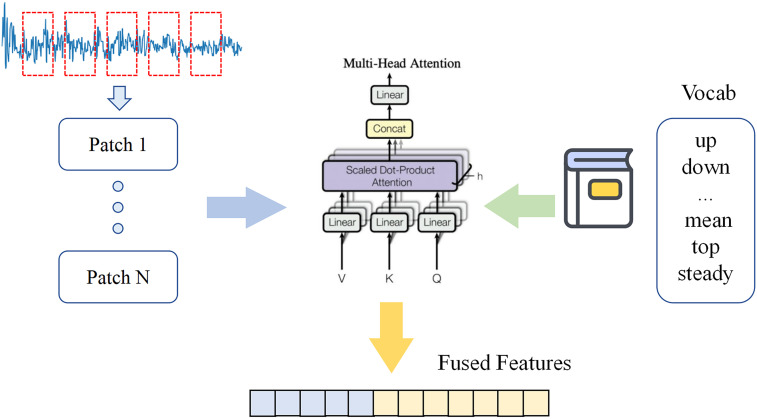
Signal-Text Alignment Module Schematic Diagram.

Vibration signal data cannot be directly edited or losslessly described using natural language, posing a significant challenge for large language models (LLMs) in understanding time-series data. The core function of the Signal-Text Modality Aligner is to establish semantic correlations between mechanical vibration signal features and natural language text, bridging the modality gap between numerical signals and linguistic symbols. The detailed structure is shown in Fig 2.

Firstly, the vibration signal Xi of length L is normalized to address the time-series distribution shift issue. Given the sliding step size of S, the input signal is partitioned into segments Patch 1 to Patch n with length T, where the total number of segments N can be determined by Equation (1).


$$N=\left[\frac{\left(L-S\right)}{T}\right]+1$$ (1)

Currently, there is no prior knowledge indicating which vocabulary terms directly correspond to specific time-series segments. Directly employing large vocabularies V for modal alignment will lead to excessive computational overhead. Therefore, domain-specific vocabulary are extracted to describe signal characteristics (e.g., “frequency-domain peaks”, “time-domain mean”, “periodic oscillations”, “rapid decline”) from a specialized dictionary, V’ with total approximately 3000 terms, V’<<V. After segmenting the time series into sliding windows, a multi-head attention mechanism is employed to perform linear combinations of these vocabulary terms. By computing the weighted relationships between signal features and word embedding vectors, semantic feature representations are generated corresponding to each time-series segment. The multi-head attention mechanism aggregates local information into each signal segment, better preserving semantic information. The detailed computational process is shown in Equations (2)-(3).


$$Multihead(Q,K,V)=Concat(head_{1},head_{2},...,head_{n})W^{h}$$ (2)

$$head_{n}=Attention(QW_{i}^{Q},KW_{i}^{K},VW_{i}^{V})$$ (3)

$W_{i}^{Q}$,$W_{i}^{K}$,$W_{i}^{V}$ is the learnable parameter matrix, and $W^{h}$ is the output transformation matrix.This model employs multiple distinct “attention heads” to compute attention in parallel, examining input information from diverse perspectives. This enables the model to capture rich and varied features/patterns within the input sequence, thereby achieving better alignment with textual features.

For our method, specifically, let the word embedding vector space in the pre-trained network be $E\in R^{V^{\prime }\times D}$, and D is the feature dimension corresponding to each word. For multi-head attention layer, each head k={1,⋯,K}, we define the query matrix $Q_{k}^{\left(i\right)}=X_{i}W_{k}^{Q}$, the key matrix $K_{k}^{\left(i\right)}=EW_{k}^{K}$, and the value matrix $V_{k}^{\left(i\right)}=EW_{k}^{V}$, where $W_{k}^{Q}\in R^{d_{m}\times d}$, and $W_{k}^{K},W_{k}^{V}\in R^{D\times d}$, $d=\left\lfloor \frac{d_{m}}{K}\right\rfloor$ We have reprogrammed each attention head.


$$Z_{k}^{\left(i\right)}=Attention(Q_{k}^{\left(i\right)},K_{k}^{\left(i\right)},V_{k}^{\left(i\right)})=Softmax(\frac{Q_{k}^{\left(i\right)}K{k\left(i\right)}^{T}}{\sqrt{d_{k}}})V_{k}^{\left(i\right)}$$ (4)

By aggregating *Z* from each head and then performing linear projection, its dimension is aligned with the dimension of the word vector space.

2. Visual encoder
**a. Time – Frequency Imaging Technology**

Since a single sensor signal cannot effectively describe all characteristics of a fault, we convert the signal time series into the time-frequency domain through time-frequency imaging techniques. This method is particularly useful in mechanical fault diagnosis because it provides an understanding of the timing of raw data and clearly reveals changes in the frequency components of the signal at different moments.

Time-frequency imaging is typically implemented using Short-Time Fourier Transform (STFT) and Continuous Wavelet Transform (CWT) techniques. The Short-Time Fourier Transform performs Fourier transform on the signal within a fixed window, making it suitable for analyzing stationary signals. In contrast, the Continuous Wavelet Transform has adaptive time-frequency resolution, enabling it to capture transient features in fault signals and making it suitable for processing complex signals containing early fault characteristics.

The mathematical expression of the CWT is as follows.


$$CWT_{x}(a,b)=\frac{\text{1}}{\sqrt{\left|a\right|}}\int_{-\infty }^{\infty }x(t)\Phi^{*}(\frac{t-b}{a})dt$$ (5)

Where x(t) is the input signal, Φ(t) is the wavelet function, a is the scale parameter, b is the translation amount, and * denotes the complex conjugate. By adjusting a and b, the CWT can explore the frequency content of the signal at different scales and time positions, providing variable time-frequency resolution, which is very suitable for capturing transient features. Through the CWT, we effectively convert one-dimensional time series data into time-frequency images, providing a richer perspective for analysis and fault diagnosis.

**b. Model Architecture**

To process time-frequency images, we employ the pre-trained CLIP visual encoder, the ViT-Base [24] model, for extracting image features. CLIP, developed by OpenAI, is a multimodal pre-trained model that learns the alignment relationship between images and text through large-scale training. Its core is to enable the model to understand the semantics corresponding to image content and establish connections with natural language descriptions. ViT-Base is a vision encoder based on the Transformer architecture, which works by dividing an image into patches, encoding these patches in a manner similar to word embedding in natural language processing, and then using the multi-head attention mechanism of Transformer to capture the relationships between image patches, thereby extracting image features.

**Fig 3:**
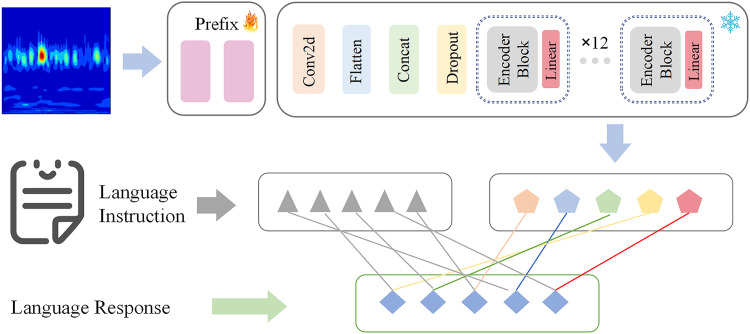
Schematic Diagram of the Visual Encoder.

As shown in Fig 3, the Visual encoder model mainly consists of three parts. The first part is the Embedding layer, which converts the input image data into a form suitable for processing by the Transformer structure using operations such as convolution and flattening. The second part is the Encoder section, which is the core module of ViT-Base. It is mainly composed of normalization layers, multi-head attention mechanisms, and Dropout layers. By stacking 12 Encoder structures, it is used to learn the features of the input image data. In addition, a linear layer is added after each Encoder layer to map the image features to the word embedding space, ensuring that this module has the same dimensionality as the text features of the language model, thus aligning with the text vectorization space. The third part is a small trainable module called the Prefix layer added at the beginning of the visual encoder to make the model better adapt to the fault diagnosis task scenario. Prefix tuning adjusts the model by adding a set of trainable virtual tokens at the front end of the network. These tokens are represented by learnable parameter vectors and participate in the self-attention calculation of the model. During training, the weights of the pre-trained model remain unchanged, and only the parameters of the Prefix layer are optimized. This method reduces computational and storage requirements and avoids the forgetting problem associated with full-model fine-tuning.

In practical applications, we use a fully connected layer as the Prefix layer, with a dimension the same as the hidden layer vector of ViT-Base. The prefix vectors and image features jointly participate in the attention calculation.


$$Attention(Q,K,V)=Softmax(\frac{Q{\left[K_{prefix},K_{input}\right]}^{T}}{\sqrt{d_{k}}})\left[V_{prefix},V_{input}\right]$$ (6)

$K_{prefix}$ and $V_{prefix}$ are the Key and Value of the prefix layer, $K_{input}$ and $V_{input}$ are the Key and Value of the input image features.

### 3. Fine-tuning module

**Fig 4:**
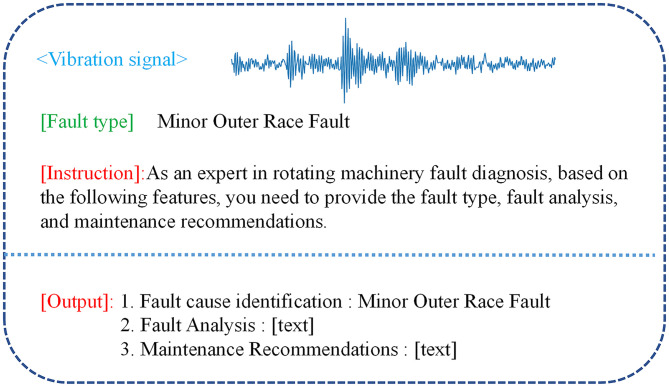
Fine-tuning datasets.

Since pre-trained large models are trained on general text databases, they inherently lack specialized vertical domain knowledge related to rotating machinery fault diagnosis. Therefore, a fine-tuning dataset for rotating machinery is constructed for fault diagnosis in the form of fault question-answer pairs. As shown in Fig 4, the vibration signals are described in numerical form, and the large model is required to output the fault type along with corresponding maintenance recommendations.

**Fig 5:**
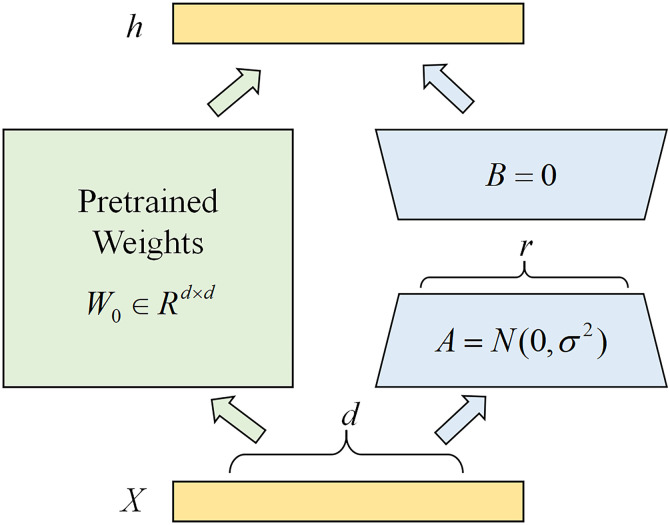
LoRA fine-tuning method.


The LoRA (Low-Rank Adaptation) method is adopted to fine-tune the large model. As shown in Fig 5, its core idea is to approximate weight updates using low-rank matrix decomposition, avoiding direct training of all the model’s parameters. The principle of parameter updates is illustrated in the Equation 7.

$$W_{0}+\Delta W=W_{0}+BA\;\;B\in R^{d\times r},A\in R^{r\times k},r<<\min(d,k)$$ (7)

The pre-trained weight matrix is denoted as $W_{0}\in R^{d\times k}$. During the training process, the parameters are frozen, and only the parameters in matrices A and B are trained.

When $h=W_{0}x$, the forward propagation process becomes $h=W_{0}x+\Delta Wx=W_{0}x+BAx$. By adopting the LoRA method, a large number of trainable parameters can be reduced, and only the introduced low-rank matrices are left for learning. Experiments have shown that despite the significant reduction in the number of parameters, LoRA can still maintain performance comparable to full fine-tuning, achieving an ideal balance between parameter efficiency and model capability.

### 4. RAG-Enhanced Retrieval Module

**Fig 6:**
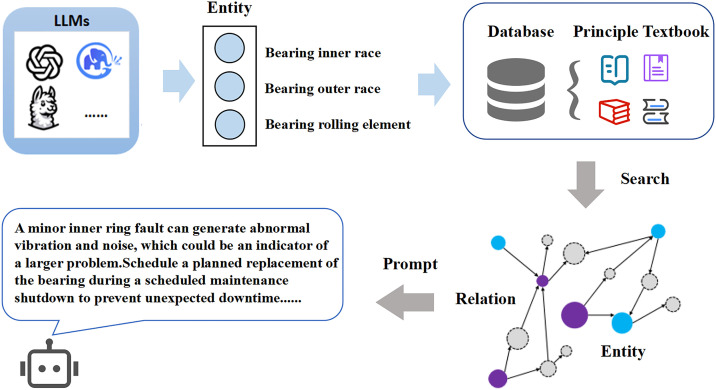
RAG-Enhanced Retrieval module.

By integrating the signal-text modality aligner, visual encoder, and fine-tuning module, the model can already effectively determine the fault type of the input vibration signal. However, since the general pre-training data of large models hardly covers the in-depth knowledge of the rotating machinery domain, the output fault causes and maintenance suggestions lack theoretical depth and professionalism. To address this issue, it is necessary to construct a vector database based on collected principle books, equipment manuals, and other materials. The specific construction process is shown in the Fig 6. Firstly, the document content is split into semantic paragraphs and remove irrelevant information such as special symbols, codes, and modal particles. And then entities and relationships within the paragraphs are extracted to form structured knowledge triples. A word embedding model is used to encode the triple information into semantic vectors for storage in the database, while index information is also stored.

During the inference process, the large language model first judges the fault type, then performs entity recognition on the fault location, thereby linking the fault location to the vector database. In the vector database, path-based retrieval and adjacency retrieval methods are adopted to obtain principle knowledge and maintenance suggestions related to the faulty component. The large language model is used to understand and arrange the text, forming a natural language maintenance assistance scheme with knowledge annotation and explanation.

## Experiments

### A. Data introduction

To verify the diagnostic accuracy and generalization ability of the model proposed in this paper, four public datasets are used for fault detection analysis under different bearing working conditions: XJTU [25], CWRU [26], HIT [27], and JNU [28]. These datasets cover multiple fault types and various working conditions. Table 1 lists the detailed sample information.

**Table 1:** Details information of datasets.

Dataset	Sample Rate(kHz)	Fault Types	Time(s)	Number of samples
**XJTU**	25.6	4	13336	1200
**CWRU**	12/48	4	3932	1200
**HIT**	20	3	9648	1200
**JNU**	100	4	3600	1200

**XJTU.** The XJTU bearing dataset, constructed by Xi’an Jiaotong University, is a public benchmark for rotating machinery fault diagnosis, collecting bearing vibration signals under various working conditions via a motor-rotor test rig.

**CWRU.** The CWRU bearing dataset from Case Western Reserve University is a standard benchmark for rotating machinery fault diagnosis. It uses accelerometers to capture vibration signals at 12kHz/48kHz, covering EDM-induced inner ring/outer ring/rolling element faults for machine learning validation.

**HIT.** The HIT bearing dataset from Harbin Institute of Technology is a public resource for rotating machinery diagnosis. Collected via a rotor test system, it uses 6205 deep-groove ball bearings with EDM-induced inner/outer ring and rolling element faults.

**JNU.** The JNU bearing dataset from Jiangnan University is for rolling bearing fault research, collected via a 3.7kW Mitsubishi induction motor system. Using roller bearings, it captures 50kHz vibration signals for normal/outer ring/rolling element faults and inner ring faults.

Due to the different signal lengths and fault type setups across datasets, we have standardized the datasets by unifying the input signal length to 12,000 and taking 2,000 samples from each dataset. The fault types are defined as minor outer ring fault, severe outer ring fault, minor inner ring fault, severe inner ring fault, and rolling element fault.

#### B. Parameter settings.

In verification experiments, 6 time-domain features and 6 frequency-domain features are selected, a total of 12 features, as the textual state description of the original input signals, enabling the large language model to better understand the time-series characteristics through semantic text information, as shown in the Table 2.

**Table 2:** Detailed information on time-frequency features.

Time domain feature	Formula	Frequency domain feature	Formula
**Mean value**	$p_{1}=\frac{\text{1}}{N}\sum {\mathrm{\backslash nolimits}}_{n=1}^{N}x(n)$	**Frequency mean value**	$p_{7}=\frac{\text{1}}{K}\sum {\mathrm{\backslash nolimits}}_{k=1}^{K}s(k)$
**Standard deviation**	$p_{2}=\sqrt{\frac{\text{1}}{N-1}\sum {\mathrm{\backslash nolimits}}_{n=1}^{N}{\left|x(n)-p_{1}\right|}^{2}}$	**Frequency variance**	$p_{8}=\frac{\text{1}}{K-1}\sum {\mathrm{\backslash nolimits}}_{k=1}^{K}\left|s(k)-p_{7}\right|$
**Absolute mean value**	$p_{3}=\frac{\text{1}}{N}\sum {\mathrm{\backslash nolimits}}_{n=1}^{N}\left|x(n)\right|$	**Gravity frequency**	$p_{9}=\frac{\sum {\mathrm{\backslash nolimits}}_{k=1}^{K}f_{k}s(k)}{\sum {\mathrm{\backslash nolimits}}_{k=1}^{K}s(k)}$
**Peak value**	$p_{4}=\max\left|x(n)\right|$	**Frequency standard deviation**	$p_{10}=\sqrt{\frac{\sum {\mathrm{\backslash nolimits}}_{k=1}^{K}{\left(f_{k}-p_{9}\right)}^{2}s(k)}{K\sum {\mathrm{\backslash nolimits}}_{k=1}^{K}s(k)}}$
**Variance**	$p_{5}=\frac{\text{1}}{N}\sum {\mathrm{\backslash nolimits}}_{n=1}^{N}{\left(x(n)\right)}^{2}$	**Frequency root mean square**	$p_{11}=\sqrt{\frac{\sum {\mathrm{\backslash nolimits}}_{k=1}^{K}f_{k}^{2}s(k)}{\sum {\mathrm{\backslash nolimits}}_{k=1}^{K}s(k)}}$
**Waveform index**	$p_{6}=p_{2}/p_{3}$	**Average frequency**	$p_{12}=\sqrt{\frac{\sum {\mathrm{\backslash nolimits}}_{k=1}^{K}f_{k}^{4}s(k)}{\sum {\mathrm{\backslash nolimits}}_{k=1}^{K}f_{k}^{2}s(k)}}$

Common evaluation metrics for fault diagnosis include ACC (Accuracy), P (Precision), R (Recall), and F1 Score. Using these metrics enables a comprehensive assessment of a model’s classification effectiveness.

**ACC.** It reflects the proportion of both faulty and normal conditions that are correctly diagnosed. The formula is defined as:


$$ACC=\frac{TP+TN}{TP+TN+FP+FN}$$ (8)

where TP represents the number of true positives (correctly identified faulty cases), TN denotes true negatives (correctly identified normal cases), FP refers to false positives (normal cases misclassified as faulty), and FN stands for false negatives (faulty cases misclassified as normal).

Precision. It reflects the ratio of actual faulty cases among all instances predicted as faulty. A higher precision indicates fewer false positive errors (false alarms). Mathematically, it is expressed as:


$$P=\frac{TP}{TP+FP}$$ (9)

F1 score. It represents the harmonic mean of precision and recall, which is particularly suitable for imbalanced datasets where the number of fault cases differs significantly from normal cases. Mathematically, it is defined as:


$$F1=\frac{2\times P\times R}{P+R}$$ (10)

Recall. It reflects the proportion of actual faults that are successfully detected. Higher recall signifies fewer instances of faulty cases being misclassified as normal. The definition is:


$$R=\frac{TP}{TP+FN}$$ (11)

The model is trained using one Nvidia A100 80G graphics card. The word embedding module adopted Bert-embedding, and the large language model used the open-source Qwen-7b. We fine-tuned Qwen-7b with the LoRA method for 8 training epochs.

### C. Experimental verification

1. Model capability comparison under the same working conditions

Under the same dataset and working conditions, the model performance is verified and compared with traditional deep learning networks including One Dimensional CNN (1D-CNN) [29], Deep CNN with Wide First – layer Kernels (WDCNN) [30], and Bidirectional Long Short-Term Memory (Bi-LSTM) [31]. The experimental results are shown in the Table 3.

**Table 3:** Performance of different models in terms of indicators.

	XJTU				CWRU			
Method	ACC(%)	P(%)	R(%)	F1(%)	ACC(%)	P(%)	R(%)	F1(%)
1D-CNN	83.36	78.33	76.68	81.78	78.93	75.32	74.23	78.95
WDCNN	91.97	89.45	86.52	88.93	92.36	90.32	86.37	89.36
Bi-LSTM	86.58	85.12	81.19	83.36	85.33	83.97	80.85	81.79
Proposed	98.34	98.65	96.87	97.52	98.72	97.25	96.33	98.97
	HIT				JNU			
1D-CNN	65.98	63.25	58.79	60.12	73.34	70.63	75.49	72.95
WDCNN	76.35	78.93	73.35	70.36	86.68	85.36	84.93	85.58
Bi-LSTM	70.43	70.69	68.57	65.25	80.37	81.32	82.35	78.51
Proposed	95.97	93.48	90.37	93.45	96.58	97.10	93.33	95.54

It can be seen that the proposed model significantly outperforms traditional deep learning models in core indicators such as ACC, R, and F1 value for bearing fault diagnosis under the same working conditions, which indicates that the proposed model can accurately capture the feature correlations in vibration signals and improve the accuracy of fault diagnosis.

### 2. Model capability comparison under the different working conditions

**Fig 7:**
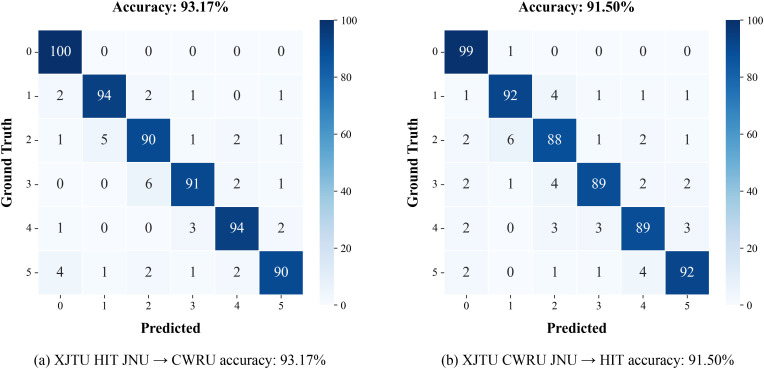
The Fault Diagnosis Performance of Our Model under Different Working Conditions.

**Fig 8:**
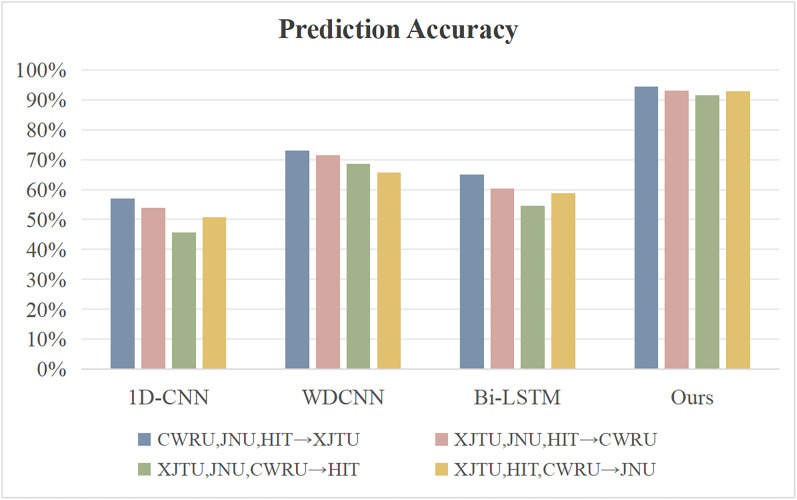
Diagnosis Accuracy of the Model under Different Working Conditions.

The generalization ability of the proposed model is verified by using three types of datasets under different working conditions as training samples to predict the fault types of the fourth type of samples. The results are shown in Figs 7-8.

When using XJTU, HIT, and JNU for training and predicting the CWRU dataset, the model’s accuracy still remains at 93.1%. In contrast, the accuracies of 1D-CNN, WDCNN, and Bi-LSTM decrease to 53.8%, 71.5%, and 60.3%, respectively. This result indicates that the proposed model can effectively capture the internal correlations of fault features under different working conditions, breaking through the dependence of traditional methods on specific working conditions. It is believed that this improvement in cross-working-condition generalization ability is mainly attributed to the semantic modeling of time-series features. Through the bidirectional context encoding mechanism, working condition parameters such as rotational speed and load are transformed into learnable semantic representations, enabling the model to maintain sensitive capture of fault features in unseen working conditions.

### 3. Impact of different modules on model performance

To quantitatively evaluate the contribution of each module to the improvement of fault diagnosis accuracy, this study employs a controlled variable approach to systematically validate the contribution levels of individual modules in cross-condition bearing fault diagnosis scenarios. The experimental results are presented in Table 4. From the experimental results, the following key conclusions can be drawn:

**Table 4:** The impact of each module on cross-condition fault diagnosis accuracy.

Signal-text Module	Visual Encorder	Word Embedding	Fine-tuned LLM	XJTU	CWRU	HIT	JNU
√			√	78.9	82.5	74.4	79.0
	√		√	72.3	75.9	68.8	72.8
		√	√	68.7	70.1	55.9	62.6
√	√		√	88.1	89.2	85.2	90.3
√		√	√	84.3	83.6	81.6	83.8
	√	√	√	78.6	80.3	71.1	77.3
√	√	√	√	94.3	93.1	91.5	92.8

**Multimodal data fusion effectively enhances fault diagnosis accuracy.** When all modules are enabled, the model achieves the highest accuracy across all four datasets compared to other combinations. This demonstrates that the synergistic integration of the Signal-Text alignment module, visual encoder, text embedding, and fine-tuned large language model fully leverages the strengths of each module, constructing a more comprehensive feature representation. The results validate the “1 + 1>2” collaborative effect of multimodal fusion, significantly improving fault diagnosis accuracy under cross-condition datasets.

**Single-module approaches exhibit inherent limitations.** Using only one module leads to significantly lower accuracy compared to full multimodal fusion. This indicates that unimodal data can not sufficiently capture the diverse features present in complex operating conditions, resulting in incomplete fault feature extraction and constrained performance. Moreover, improper module combinations (e.g., pairing the visual encoder with word embeddings while omitting direct vibration signal information) may introduce feature redundancy or encoding conflicts, impairing the model’s ability to learn discriminative fault characteristics. For instance, certain combinations achieved only 50%−60% accuracy, highlighting the necessity of rational module compatibility for optimal performance.

**The Signal-Text alignment module and LLM jointly form the foundation of the model.** Combinations incorporating these two components consistently yield higher accuracy. This underscores that raw vibration signals provide essential fault-related information, while their absence leads to fragmented and poorly correlated features, hindering the model’s ability to link operational conditions with fault logic.

**Model generalization relies on multimodal data.** Under full-module fusion, the accuracy gap across the four datasets is minimal, indicating that multimodal collaboration ensures balanced adaptability to varying operational conditions. In contrast, partial module combinations exhibit larger performance fluctuations, revealing their limited robustness to cross-condition variations. These observations further confirm that multimodal fusion strengthens the model’s cross-domain generalization capability.

### 4. The performance of the model in fault diagnosis question answering

**Fig 9:**
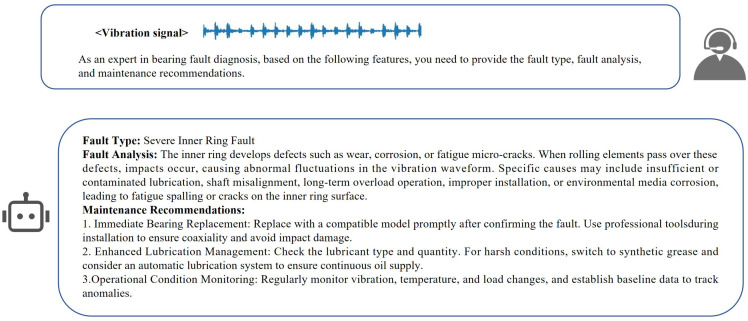
Answers generated by general large language models.

**Fig 10:**
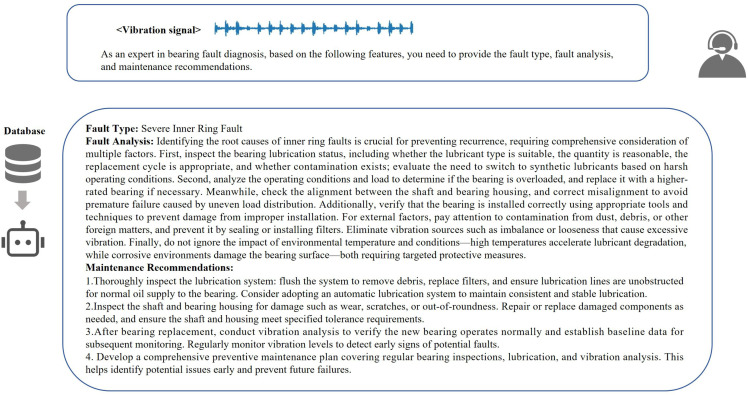
Responses generated by large language model with external knowledge base.

This part focuses on evaluating the content generation effect of the model in the fault diagnosis Q&A scenario. By comparing the quality differences of the model’s answers when the knowledge base is enabled/disabled, we verify whether the external knowledge base can effectively make up for the professionalism gap of the model native knowledge in the industrial field. The answer content is shown in Figs 9-10.

From the generated content, it can be seen that the answers based on the external knowledge base have more advantages than the basic version. In terms of fault analysis, it is no longer limited to superficial associations, but refines the failure link and upgrades from phenomenon description to root cause dissection. In terms of maintenance suggestions, compared with the answers of the basic model, our model constructs a prevention system, covering the whole – process schemes such as bearing replacement technology, lubrication management, and shafting monitoring, and the answer content is more professional. However, due to the lack of evaluation indicators, a quantifiable standard system has not been formed at present. It is difficult to accurately measure the advantages and disadvantages of the content generated by the large model in dimensions such as the depth of fault mechanism analysis and the practicality of maintenance suggestions. We can only rely on subjective human judgment to determine the quality of the generated content, and cannot objectively determine the specific value of its contribution to the industrial operation and maintenance scenario. In the future, we need to strengthen the fine – grained evaluation and continuous optimization of the large model’s ability to assist in bearing fault diagnosis.

## Conclusions

To sum up, our study addresses the inherent limitations of traditional single-modal vibration signal analysis, namely, the difficulty in fully covering the fault feature space of complex mechanical systems and the insufficient cross-condition generalization ability. By proposing an intelligent diagnosis framework based on multimodal large models, a ternary data system of “vibration time-domain signals—time-frequency spectrum features—fault knowledge text” is constructed to realize the cross-modal joint representation of mechanical fault features, breaking through the bottlenecks of traditional methods such as insufficient feature extraction capability under complex working conditions and limited cross-scene adaptability. The framework innovatively integrates the deep semantic understanding ability of pre-trained large language models with mechanical fault mechanisms. Through the method of embedding a principle-based knowledge base, the model can not only accurately locate fault positions but also simultaneously generate interpretable reports including fault cause analysis and maintenance strategy recommendations.

Looking to the future, this research lays a foundation for the subsequent development of compound fault representation and intelligent diagnosis, as well as the expansion of multimodal large models to broader industrial PHM applications. By bridging the gap between time-series data and text semantics, it promotes the transformation of traditional fault diagnosis toward more interpretable, adaptive, and professional solutions.
